# Generating low immunogenic pig pancreatic islet cell clusters for xenotransplantation

**DOI:** 10.1111/jcmm.15136

**Published:** 2020-03-25

**Authors:** Marco Carvalho Oliveira, Emilio Valdivia, Murielle Verboom, Yuliia Yuzefovych, Hendrik Johannes Sake, Olena Pogozhykh, Heiner Niemann, Reinhard Schwinzer, Björn Petersen, Jochen Seissler, Rainer Blasczyk, Constança Figueiredo

**Affiliations:** ^1^ Institute for Transfusion Medicine Hannover Medical School Hannover Germany; ^2^ Transregional Collaborative Research Centre 127 Munich Germany; ^3^ Department of Biotechnology Institute of Farm Animal Genetics Friedrich‐Loeffler‐Institute Federal Research Institute for Animal Health Greifswald‐Insel Riems Germany; ^4^ Clinic for Gastroenterology, Hepatology and Endocrinology Hannover Medical School Hannover Germany; ^5^ Transplantation Laboratory Clinic for General Visceral and Transplantation‐Surgery Hannover Medical School Hannover Germany; ^6^ Diabetes Center Medizinische Klinik und Poliklinik IV Klinikum der Universität München Munich Germany

**Keywords:** immunogenicity, islet‐like cell clusters, RNAi, SLA class I and class II silencing, xenotransplantation

## Abstract

Xenotransplantation of pancreatic islets offers a promising alternative to overcome the shortage of allogeneic donors. Despite significant advances, either immune rejection or oxygen supply in immune protected encapsulated islets remains major bottlenecks for clinical application. To decrease xenogeneic immune responses, we generated tissue engineered swine leucocyte antigen (SLA)‐silenced islet cell clusters (ICC). Single‐cell suspensions from pancreatic islets were generated by enzymatic digestion of porcine ICCs. Cells were silenced for SLA class I and class II by lentiviral vectors encoding for short hairpin RNAs targeting beta2‐microglobulin or class II transactivator, respectively. SLA‐silenced ICCs‐derived cells were then used to form new ICCs in stirred bioreactors in the presence of collagen VI. SLA class I silencing was designed to reach a level of up to 89% and class II by up to 81% on ICCs‐derived cells. Xenogeneic T cell immune responses, NK cell and antibody‐mediated cellular‐dependent immune responses were significantly decreased in SLA‐silenced cells. In stirred bioreactors, tissue engineered islets showed the typical 3D structure and insulin production. These data show the feasibility to generate low immunogenic porcine ICCs after single‐cell engineering and post‐transduction islet reassembling that might serve as an alternative to allogeneic pancreatic islet cell transplantation.

## 1 INTRODUCTION

Diabetes mellitus is characterized by chronic hyperglycaemia caused by abnormalities in insulin secretion, action or both. Eighty to 90% of the cases of diabetes in children and adolescents are suffering from type 1 diabetes resulting from complete autoimmune destruction of pancreatic β‐cells through cellular immune responses.^1^, ^2^, ^3^ Human allogeneic pancreas transplantation represents a successful treatment of type 1 diabetes. Nevertheless, because of its complexity and the risks associated, such as formation of blood clots, resurgence of pancreatitis and undesired side‐effects from the immunosuppressive treatments, it is not considered as routine therapy.^4^, ^5^ Human allogeneic transplantation of encapsulated islets has shown promising results to treat type 1 diabetes. However, the scarcity of islet donors and the increasing incidence of type 1 diabetes pose a relevant hurdle to allogeneic islet transplantation. Hence, xenotransplantation may offer a viable and sustainable alternative to the transplantation of the rare human cells. Nevertheless, xenogeneic organs and tissues can trigger humoural and cellular immune responses. In the case of xenogeneic islet transplantation, instant blood‐mediated inflammatory reaction mainly involving complement activation and platelet aggregation may lead to rapid loss of more than 50% of the β‐cells.^6^, ^7^, ^8^ Moreover, rejection of the xenograft may be mediated by concerted humoural and cellular immune responses, mainly characterized by pro‐inflammatory cytokines and oxygen‐reactive species derived from neutrophils. Simultaneously, xenoantibodies recognize key molecules such as MHC class I antigens and activate antibody‐dependent cell‐mediated cytotoxicity responses by natural killer (NK) cells.^9^, ^10^, ^11^

Encapsulation devices have been utilized to form an immunobarrier which possess two main characteristics: immunoprotection against immune‐competent cells including T cells, B cells or macrophages, antibodies and complement; and compatible with diffusion of glucose, nutrients and insulin. However, the lack of adequate oxygen supply to preserve the function and viability of the encapsulated islets is the most crucial obstacle for standardized clinical application of this technology. This problem is further exacerbated by the required high numbers of islets to maintain normoglycemia.^12^, ^13^, ^14^, ^15^

Tissue genetic engineering offers the possibility to modify the graft properties to improve graft survival. Previously, we have demonstrated the feasibility to efficiently and stably down‐regulate MHC class I and class II antigens in different cell types such as megakaryocytes^16^ and hepatocytes as well as in the original 3D structure of complex tissues and organs such as the corneas or lungs. Downregulation of MHC expression was compatible with immunological tolerance and supported survival of allografts by preventing humoural and cellular immune responses.^17^, ^18^ In the present study, we have combined gene therapy and tissue engineering strategies to generate porcine pancreatic islets with low immunogenicity. Collagen has supported engineered tissue development and application because of its biocompatible properties, availability, low antigenicity and biodegradability.^19^, ^20^ Hence, we investigated the potential of silencing SLA class I and SLA class II on pig islet‐derived β‐cells towards reduction of xenogeneic immune responses. In addition, we developed a method to reassemble the islets after single‐cell engineering supported by collagen matrices, leading to the reacquisition of its originals 3D structure and mimicking the original microenvironment of the islets of Langerhans in the pancreas.

## 2 MATERIALS AND METHODS

### 2.1 Animals

Ten pancreata were harvested from 2‐ to 5‐day‐old wild‐type Landrace pigs and transported at 4°C to the laboratory within 60 minutes for islet isolation and purification. All animals were maintained according to the German animal welfare law.

### 2.2 Islet‐like cell clusters and monolayer cells isolation

Pancreata were minced, and islet‐like cell clusters (ICCs) were then isolated using collagenase P enzyme (Roche) and purified using a Ficoll 400 gradient (Sigma‐Aldrich). Viable islets were handpicked under a stereomicroscope (Leica) using a pipette (Eppendorf). Isolation of ICCs‐derived cells was performed using a trypsin solution (0.25% in PBS) for 10 minutes at 37°C and 5% CO_2_. Afterwards, the cells were aspirated using a 18‐gauge cannula and cultured in RPMI‐1640 (Lonza), supplemented with 10% fetal calf serum, 10 ng/mL human epidermal growth factor (PeproTech) and 1% penicillin/streptomycin (Gibco, Thermo Fisher Scientific).

### 2.3 DTZ staining

To determine the purity of insulin‐producing β‐cells, dithizone staining (Merck Millipore) was performed. The cells were stained with DTZ enzymatic reaction for 30 minutes at 37°C, and using an optical microscope (Olympus), the pancreatic insulin‐producing beta cells became visible by crimson red staining.

### 2.4 Generation of SLA‐silenced pancreatic cells

ICCs‐derived cells from four different donors per group were seeded in a six‐well adherent plate with fresh medium and maintained until 80% confluency was reached. Cell transduction was performed in the presence of 2.5 × 10^9^ lentiviral particles together with 8 mg/mL protamine sulphate (Sigma‐Aldrich) at 37°C overnight. After incubation, cells were washed and cultured with fresh medium.

### 2.5 Live/dead cell viability assay

After overnight cell transduction, ICCs‐derived cells from four different donors per group were resuspended and viability was determined by propidium iodide (Biolegend) staining according to the manufacturer's guidelines. Propidium iodide‐positive cells were considered necrotic.

### 2.6 Evaluation of SLA class I and class II silencing effect

#### 2.6.1 Flow cytometric analysis

To evaluate SLA‐I silencing effects from four donors per each group, non‐transduced (NT), transduced with a non‐specific shRNA (shNS) and SLA‐I silenced (shβ2M) ICCs‐derived cells were stained with anti‐SLA class I‐unconjugated antibody (JM1E3, Bio‐Rad) and goat anti‐mouse IgG conjugated with PE as a secondary antibody (Biolegend). Regarding SLA‐II silencing effect, NT, non‐specific transduced (shNS) and SLA‐II silenced (shCIITA) ICCs‐derived cells were stained with anti‐SLA class II DQ (K274.3G8)‐unconjugated antibody (Bio‐Rad) and goat anti‐mouse IgG conjugated with PE as a secondary antibody (Biolegend). All samples were then analysed by flow cytometry. Transduction efficiency was determined by the percentage of GFP‐positive cells and the mean fluorescence intensity (MFI) measured by the PE secondary antibody representing the level of SLA‐I or SLA‐II expression on the cell membrane.

#### 2.6.2 Real‐time polymerase chain reaction

β2‐Microglobulin (SLA‐I) and DRA (SLA‐II) mRNA levels of pig ICCs‐derived cells were analysed by quantitative PCR. Cells were harvested, and total RNA was isolated using the RNeasy Mini Kit (Qiagen) according to the manufacturer's instructions. To transcribe total RNA into cDNA, a High‐Capacity cDNA Reverse Transcription Kit was used (Applied Biosystems). β2M and DRA transcript levels were analysed by real‐time PCR using specific gene expression assays (Ss03391156_m1 and Ss03389945_m1, respectively; Thermo Fisher). Each sample was run in duplicate. Expression levels were normalized to GAPDH (Ss03375629_u1; Thermo Fisher).

### 2.7 T cell degranulation assay

CD3 T cells were isolated from four healthy donors after informed consent. Briefly, buffy coats were diluted 1:2 with phosphate buffered saline (PBS) and centrifuged under a density gradient in Lymphosep (C. C. Pro). T cells were then isolated by magnetic‐activated cell sorting according to the manufacturer's guidelines (Miltenyi Biotec) using a negative selection antibody cocktail. CD3 T cells were primed for 8 days with ICCs‐derived NT target cells (1:5 target:effector ratio) on a 96‐well flat‐bottom plate (TPP). On day 8, primed CD3 T cells were cultured with genetically engineered ICCs‐derived cells from 4 different animals per group (1:2 and 1:5 T:E ratio) on a 96‐well U‐bottom plate (Falcon) for 6 hour at 37°C on a humidified incubator. Supernatant was collected, and cells were stained with CD3‐PerCP and CD107a‐PE (Biolegend) for FACS analysis. SLA‐silenced samples (shβ2M and shCIITA) were compared against shNS and NT.

### 2.8 NK cell degranulation assay

Human NK cells were isolated from eight healthy donors after informed consent. Briefly, buffy coats were diluted 1:2 with PBS and centrifuged under a density gradient in Lymphosep (C. C. Pro). NK cells were isolated by magnetic‐activated cell sorting according to the manufacturer's instructions (Miltenyi Biotec Bergisch) using a negative selection antibody cocktail and cultured on a 96‐well U‐bottom plate (Falcon, Corning Brand) with complete RPMI‐1640 medium (Lonza), supplemented with 5% human serum (C. C. Pro) and 100 U IL‐2 (PeproTech). The cells were maintained overnight at 37°C in 5% CO_2_ at density of 1 × 10^6^ cells/mL. ICCs‐derived cells from four animals per group were cultured with isolated NK cells (1:2 and 1:5 T:E ratio) on a 96‐well U‐bottom plate (Falcon) for 4 hour at 37°C. Supernatant was collected, and cells were stained with CD3‐PerCP, CD56‐AF647 and CD107a‐PE (Biolegend) for FACS analysis. SLA‐silenced samples (shβ2M and shCIITA) were compared against shNS and NT.

### 2.9 Antibody‐dependent cellular cytotoxicity

Genetically engineered SLA‐silenced ICCs‐derived cells from four different donors per group were harvested and seeded on a white flat 96‐well plate at day zero. At day 1, different antibody concentrations were added together with Jurkat effector cells from ADCC Reporter Bioassay Kit (Promega) with an effector:target ratio of 6:1. Cells were incubated for 6 hours at 37°C in a humidified CO_2_ incubator. After incubation, plates were taken from the incubator and a Bio‐Glo Luciferase Assay Reagent was added. The levels of bioluminescence were measured using a luminometer (Berthold Technologies GmbH).

### 2.10 Generation of SLA‐silenced porcine pancreatic islets

For reaggregation into ICCs, SLA‐engineered pancreatic cells from three donors per group were resuspended in growth medium and supplemented with 10 mmol/L nicotinamide (Sigma‐Aldrich) and 20 nmmol/L exendin‐4 (Sigma‐Aldrich).^21^ Cells were seeded at density of 5 × 10^5^ cells/mL in 100‐mL spinner flasks (Pfeiffer electronic engineering GmbH) in the presence of 10 µg/mL native human collagen type VI protein (Abcam), at 37°C in 5% CO_2_ incubator at 50 rpm. Every second day, culture medium was changed, and seven days after culture, SLA‐engineered islets were fully formed.

### 2.11 In vitro insulin release assay

Glucose‐stimulated insulin secretion assay was performed after reaggregation of SLA‐engineered pancreatic cells from three different animals per group. ICCs were handpicked, washed by incubation for 30 minutes at 37°C in Krebs‐Ringer Bicarbonate (KRB) buffer without any glucose and then incubated for 1 hour at 37°C in KRB buffer containing 20 mmol/L glucose (Sigma‐Aldrich). ICCs were collected, and total RNA was isolated using RNeasy Mini Kit (Qiagen) according to the manufacturer's guidelines. In order to transcribe the total RNA into cDNA, a High‐Capacity cDNA Reverse Transcription Kit was used (Applied Biosystems). Insulin transcript levels were analysed by real‐time PCR using specific gene expression assays (Ss03386682, Thermo Fisher). Each sample was run in duplicate. Expression levels were normalized to GAPDH (Ss03375629_u1; Thermo Fisher).

### 2.12 Statistical analysis

All statistical analysis was performed using GraphPad Prism 5 software (Graphpad Software). Results were demonstrated as mean ± standard deviation (SD). Analyses between three or more groups were performed by one‐way ANOVA, followed by Tukey's secondary test for significance. Differences were considered significant at *P* < .05.

## 3 RESULTS

### 3.1 Genetic engineering of ICCs‐derived cells

**Figure 1:**
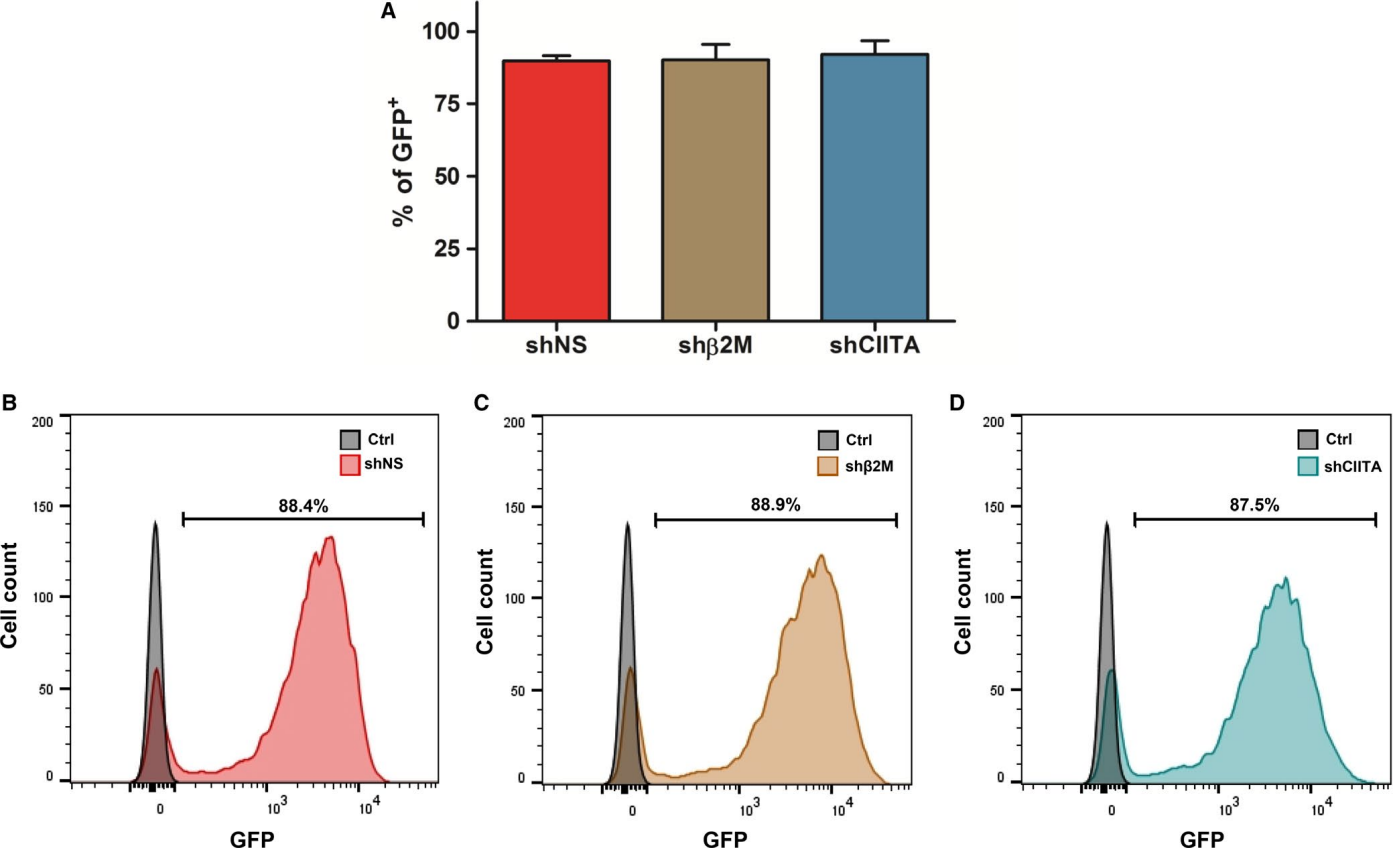
Islet cell clusters (ICCs)‐derived cells are efficiently transduced by lentiviral vectors. To overcome the problem of low transduction efficiencies of ICCs because of their compact 3D structure, those were dissociated and the isolated cells were cultured as monolayer. A, Isolated cells were transduced with lentiviral vectors encoding for swine leucocyte antigen (SLA)‐specific short hairpin RNAs (shRNAs; shNS, shβ2M, shCIITA) and GFP as a reporter gene. Three days after transduction, cells were collected and the percentages of GFP‐positive cells were analysed by flow cytometry and compared to the control NT cells. Graph shows means and standard deviations of four independent donors in each condition. B, Representative histogram of GFP‐positive cells transduced with shNS‐, C, shβ2M‐ and D, shCIITA‐encoding lentiviral vector

Efficient genetic modification of isolated porcine pancreatic islets remains challenging because of its 3D complex and compact structure.^22^ Therefore, the genetic modification of isolated ICCs‐derived cells in monolayer may offer a more efficient alternative. To reduce the immunogenicity of ICCs‐derived cells, lentiviral vectors encoding GFP as reporter gene were used (Figure 1). Flow cytometric analysis for the detection of GFP expression demonstrated similar transduction efficiencies with all three different vectors (Figure 1A). Control shNS cells were transduced by 89.8 ± 1.9% (Figure 1B), while shβ2M (Figure 1C) and shCIITA (Figure 1D) cells were transduced by 90.2 ± 5.2% and 92.1 ± 4.6%, respectively. These data demonstrate the feasibility to efficiently transduce ICCs‐derived cells.

### 3.2 Silencing SLA class I and SLA class II transcript and protein expression levels in ICCs‐derived cells

**Figure 2:**
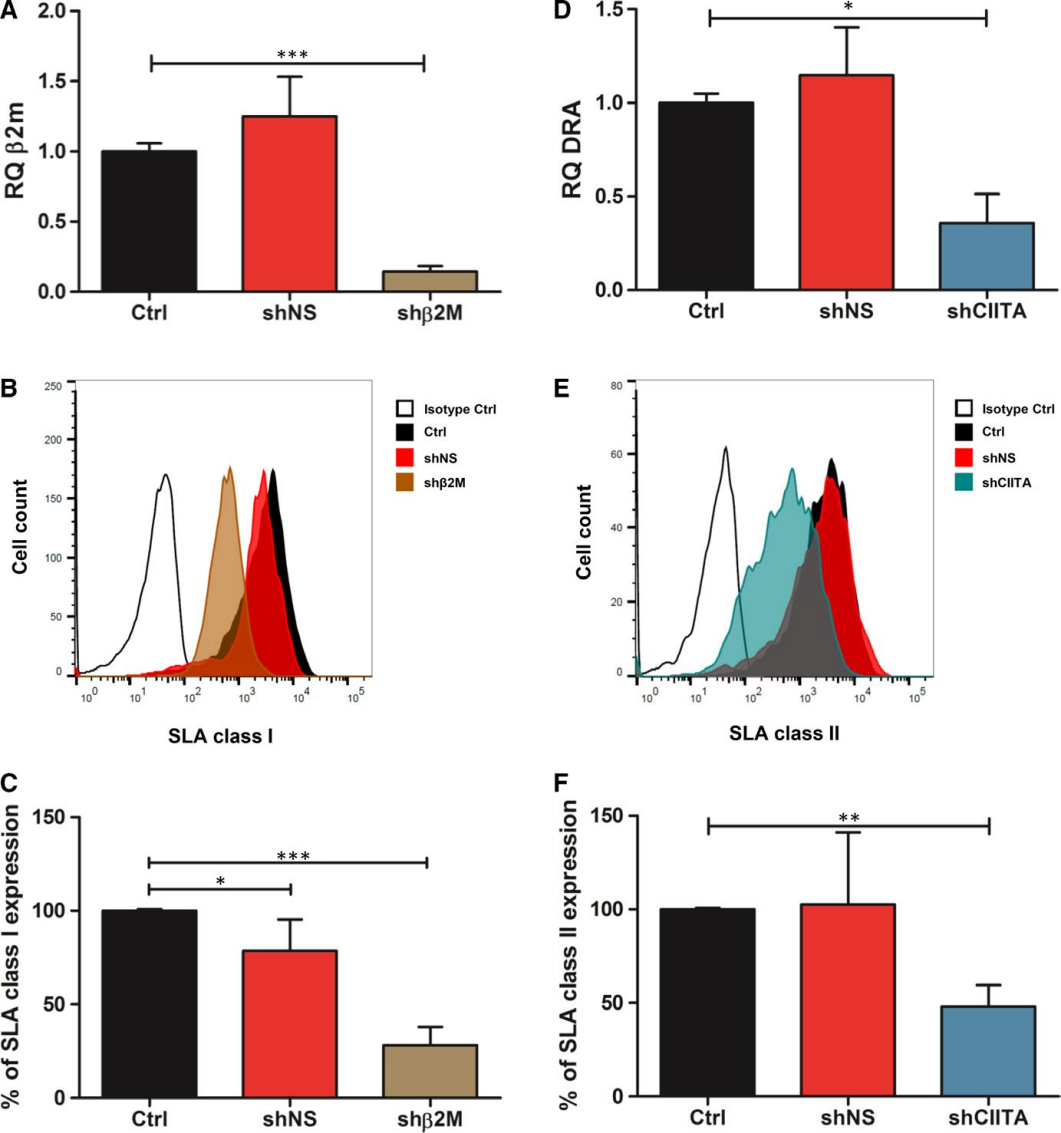
Silencing SLA class I and class II expression in ICCs‐derived cells. A, Relative quantification (RQ) of β2M detected in shNS and shβ2M‐expressing ICC‐derived cells stimulated with interferon gamma (IFN‐γ) and tumour necrosis factor alpha (TNF‐α). B, Representative histogram plots of SLA class I expression on ICCs‐derived cells transduced with lentiviral vectors. NT ICCs‐derived cells or shNS cells were used as controls. C, Mean and standard deviations of percentages of SLA class I expression. D, RQ values of DRA transcript levels detected in shNS‐ and shCIITA‐expressing ICC‐derived cells stimulated with IFN‐γ and TNF‐α. E, Representative histogram plots of SLA class II expression. NT ICCs‐derived cells or shNS cells were used as controls. F, Mean and standard deviations of SLA class II expression levels. Percentage of expression was calculated by normalizing the MFI to those measured in the respective control sample. Transcript levels were normalized to the endogenous control gene GAPDH. Graph shows means and standard deviation of ≥4 independent assays. Statistical significance was calculated by using one‐way ANOVA followed by Tukey's post hoc test. *P < .05; **P < .01; ***P < .001

Major and minor histocompatibility antigens define tissue identity in transplantation and trigger immune responses that may lead to graft rejection.^23^ ICCs‐derived cells transduced with shβ2M‐encoding vectors resulted in downregulation of β2M transcripts levels by up to 89% (*P* < .001) in comparison with control cells (Figure 2A). This contributed to the decrease in SLA class I cell surface proteins of 72% (*P* < .001) on ICCs‐derived cells in comparison with NT control cells (Figure 2B,C). Similarly, delivery of shCIITA‐encoding vectors to ICCs‐derived cells reduced by up to 81% (*P* < .05) of DRA transcript levels when compared to control cells (Figure 2D). At protein level, flow cytometric analysis revealed a downregulation by up to 69% (*P* < .01) of SLA class II expression in shCIITA‐expressing cells (Figure 2E,F). NT control cells and shNS showed comparable SLA class I and class II transcript and protein expression levels. These data demonstrate the feasibility to directly regulate SLA class I and class II gene and protein expression after vector‐mediated shRNA delivery in ICC‐derived cells.

### 3.3 Silencing SLA class I and SLA class II does not impair cell viability

**Figure 3:**
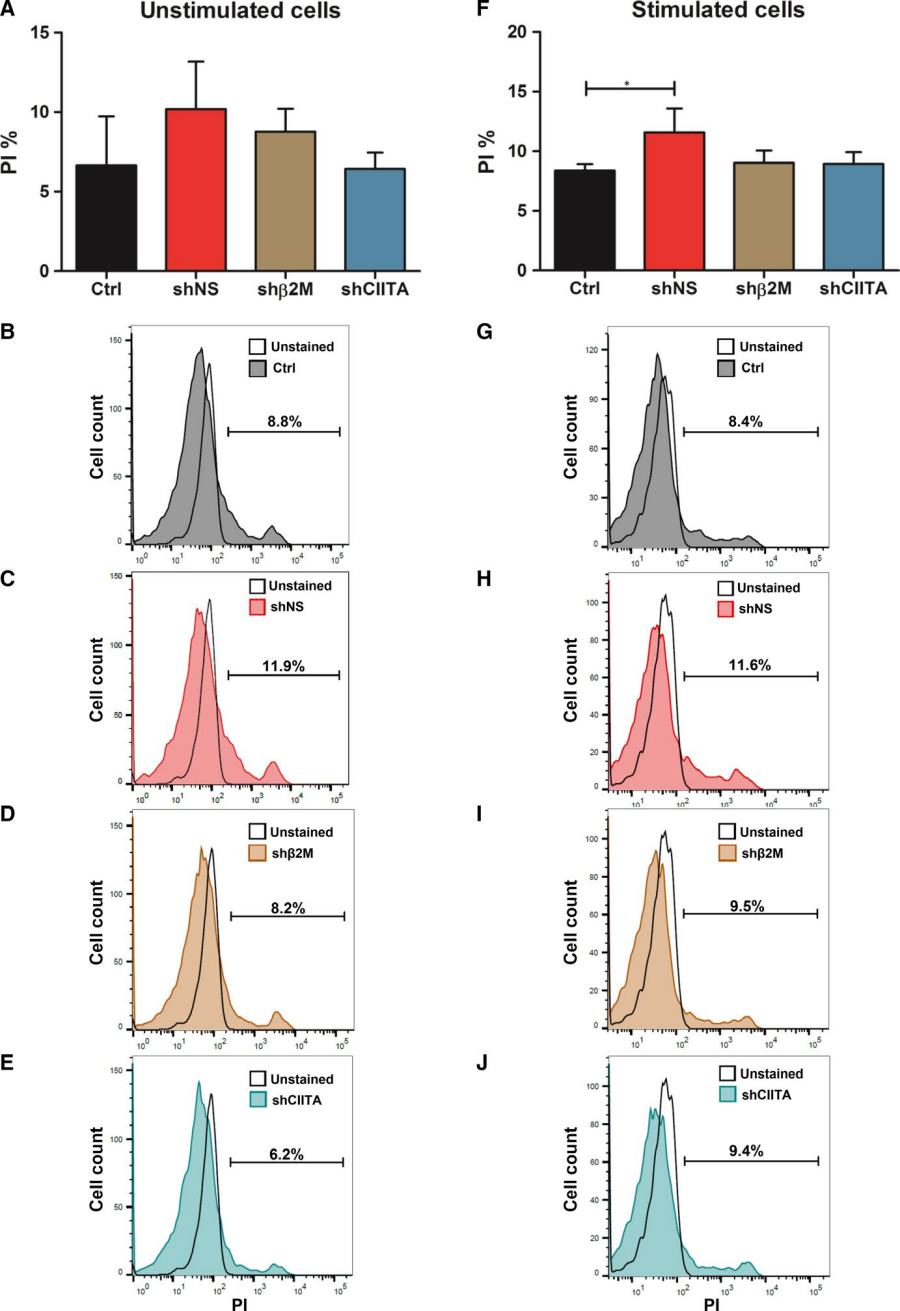
Silencing SLA expression does not affect cell viability. A, Percentage of propidium iodide (PI)‐stained cells are displayed as mean and SD in the bar graphs without and F, with IFN‐γ and TNF‐α stimulation (*P < .05). B, Representative overlay histograms of PI‐stained control (Ctrl) cells, C, shNS‐expressing cells, D, shβ2M‐ and E, shCIITA‐silenced cells without stimulation with IFN‐γ and TNF‐α compared to unstained Ctrl cells. G, Representative histogram plots of PI‐stained Ctrl cells, H, shNS‐transduced cells, I, shβ2M‐ and J, shCIITA‐silenced cells stimulated with IFN‐γ and TNF‐α compared to the respective unstained control cells. Graphs depict means and standard deviations, n = 4. Statistical significance was calculated using one‐way ANOVA followed by Tukey's post hoc test. *P < .05

Quality of genetic engineered ICC‐derived cells is crucial to ensure their function. Hence, we compared the effects of lentiviral transduction using the vectors encoding for shNS, shβ2M and shCIITA in cell viability (Figure 3). No significant differences were observed between the control NT ICCs‐derived cells and the three different genetically engineered cells without IFN‐γ and TNF‐α stimulation. Cells stained positively with PI ranged between 10.2 ± 3.0% in shNS, 8.8 ± 1.4% in shβ2M and 6.4 ± 1.0% in shCIITA compared to 6.7 ± 3.0% in the control (Ctrl). In addition, we measured cell viability also in the presence of IFN‐γ and TNF‐α to mimic a pro‐inflammatory environment. A slight but significant increase (*P < *.05) in cell necrosis was detected in shNS‐expressing cells after IFN‐γ stimulation compared to the control NT cells, 11.6 ± 2.0 to 8.4 ± 0.5%, respectively. ICCs‐derived cells transduced with shβ2M‐encoding vector, 9.0 ± 1.0%, and shCIITA, 9.0 ± 1.0%, did not show significant differences in comparison with the control cells. These data show that silencing SLA expression does not impair cell viability.

### 3.4 Silencing SLA class II decreases T cell degranulation

**Figure 4:**
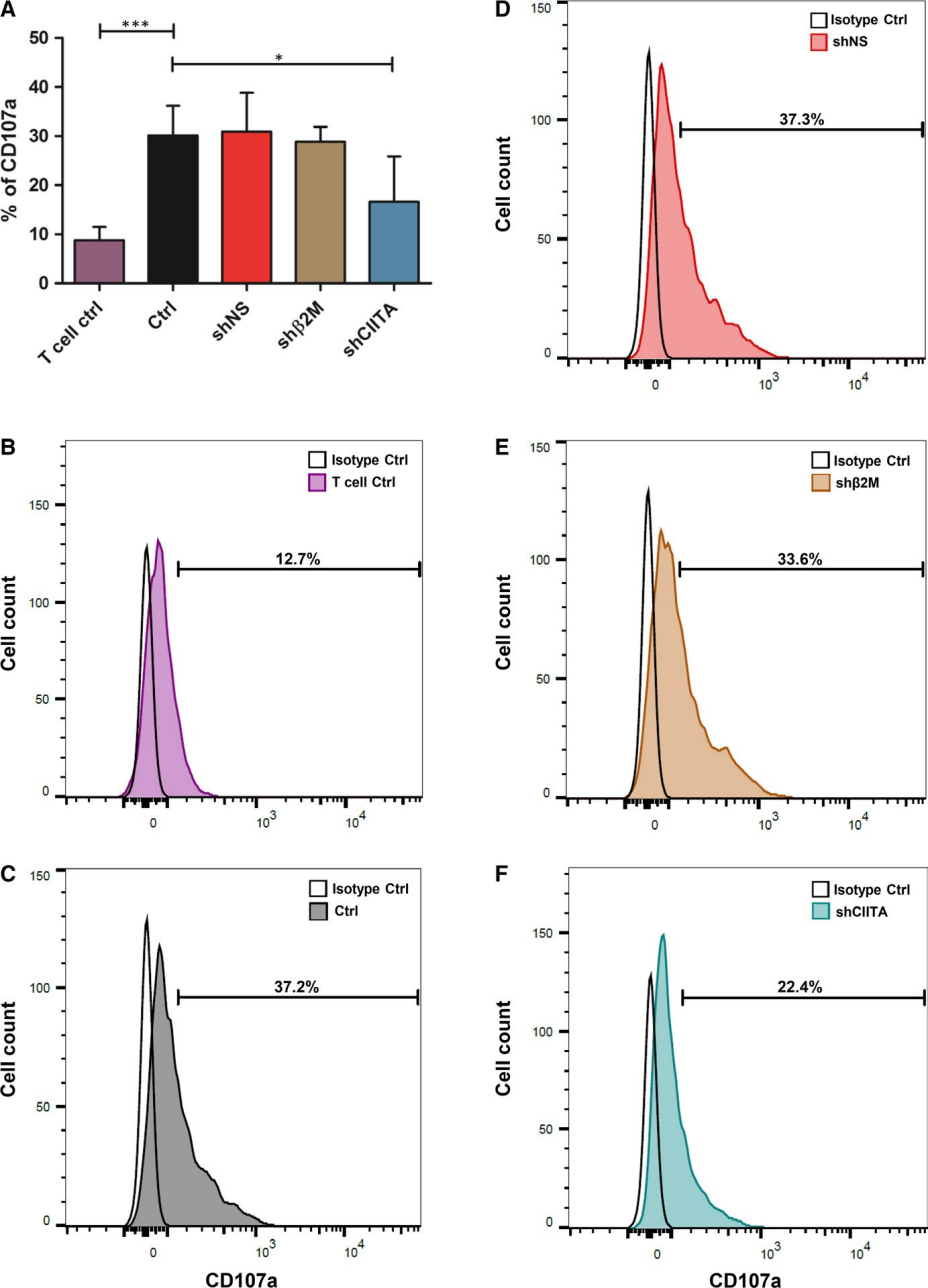
Effect of SLA silencing in the T cell xenogeneic immune response. Primed T cells were co‐cultured with SLA‐silenced ICCs‐derived cells for 8 h, and T cell degranulation levels were measured by detecting CD107a expression. A, Bar graph shows means and standard deviations of CD107a expression determined by flow cytometry analysis. B, Representative overlay of spontaneous T cell degranulation (T cells only). C, Representative histogram plot of CD107a expression on T cells culture with Ctrl cells, D, shNS‐, E, shβ2M‐ and F, shCIITA‐silenced cells. Statistical significance was calculated using one‐way ANOVA followed by Tukey's post hoc test. *P < .05; ***P < .001

T cell recognition of graft's antigens may contribute to their activation and degranulation leading to rejection. Therefore, the effect of MHC silencing on T cell degranulation was evaluated by measuring CD107a molecules (Figure 4). We did not observe any significant difference in T cell degranulation in co‐culture experiment with shβ2M‐expressing cells (28.8 ± 3.1%) in comparison to control cells (Ctrl) and shNS, 30.1 ± 6.0% and 30.9 ± 8.0%, respectively. Interestingly, SLA class II‐silenced cells induced significantly (*P* < .05) decreased means of 16.6 ± 9.0% degranulating T cells in comparison with control cells. These data demonstrate that SLA class II‐silenced ICC‐derived cells are protected against T cell responses.

### 3.5 Silencing SLA class I expression does not increase the susceptibility to NK cell activity

**Figure 5:**
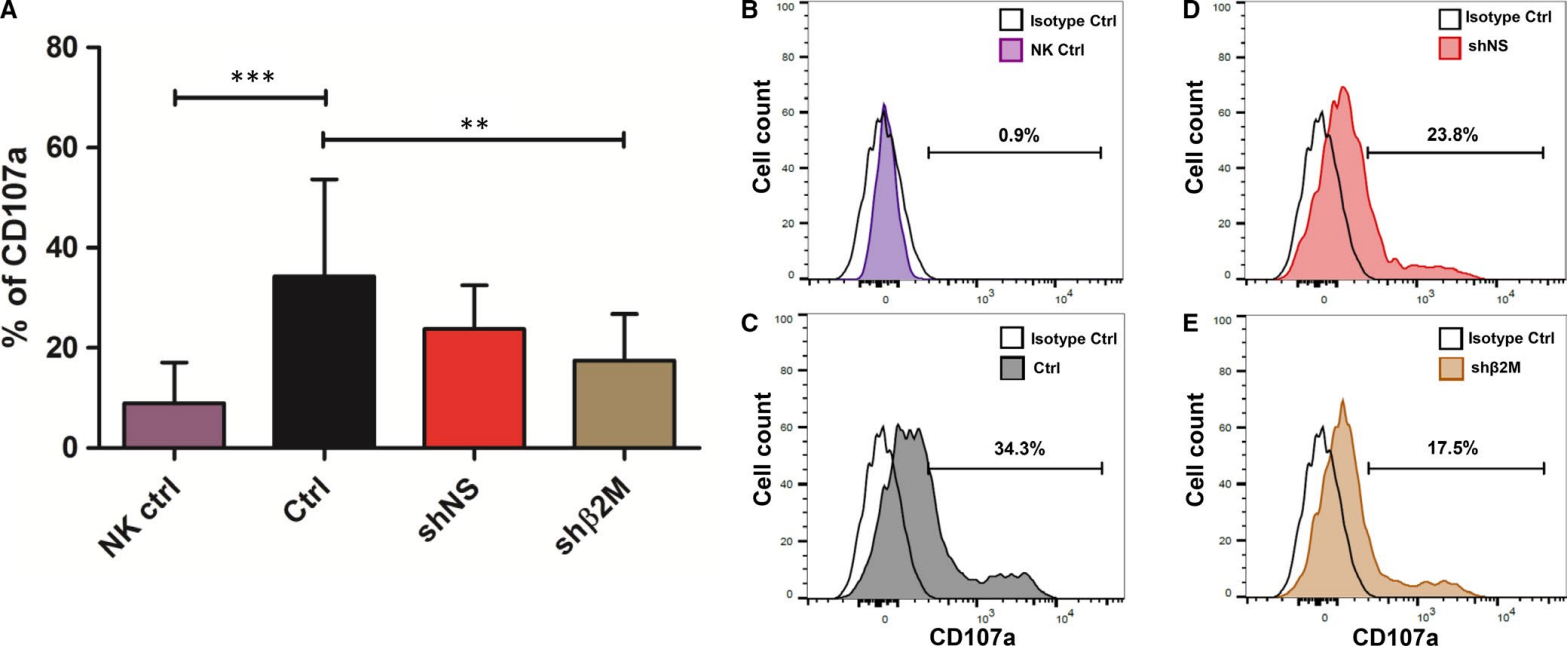
Effect of SLA class I silencing effect on NK cell xenogeneic immune response. ICCs‐derived cells were co‐culture with NK cells for 4 h, and degranulation was measured by flow cytometry. A, Levels of CD107a expressed in NK cells cultivated alone (NK Ctrl), control NT cells (Ctrl) and from genetically engineered ICCs‐derived cells (shNS and shβ2M). B, Representative histogram plots from spontaneous NK cell degranulation, C, NK cell degranulation towards control NT ICCs‐derived cells, D, CD107a expression after NK cell coculture with shNS cells and E, NK cell co‐culture with SLA class I‐silenced ICCs‐derived cells. Statistical significance was calculated using one‐way ANOVA followed by Tukey's post hoc test. **P < .01; ***P < .001

In a transplantation setting, MHC class I antigen recognition by killer cell immunoglobulin‐like receptors plays a key role in distinguish self from non‐self cells.^24^ Therefore, we analysed NK cells degranulation levels upon silencing of SLA class I molecules (Figure 5). Interestingly, a significant (*P* < .01) decrease of CD107a levels obtained from co‐culture of human NK cells with porcine shβ2M‐expressing ICC‐derived cells was observed when compared to the control cells, 17.5 ± 9.3% to 34.3 ± 19.4%, respectively. There was no significant difference in NK cell degranulation levels for control and shNS‐expressing ICC‐derived cells, 34.3 ± 19.4% versus 23.8 ± 8.7%, respectively. These data show that silencing SLA class I expression does not trigger xenogeneic NK cell activation.

### 3.6 Silencing SLA class I and SLA class II expression protects against antibody‐dependent cellular‐mediated cytotoxicity

**Figure 6:**
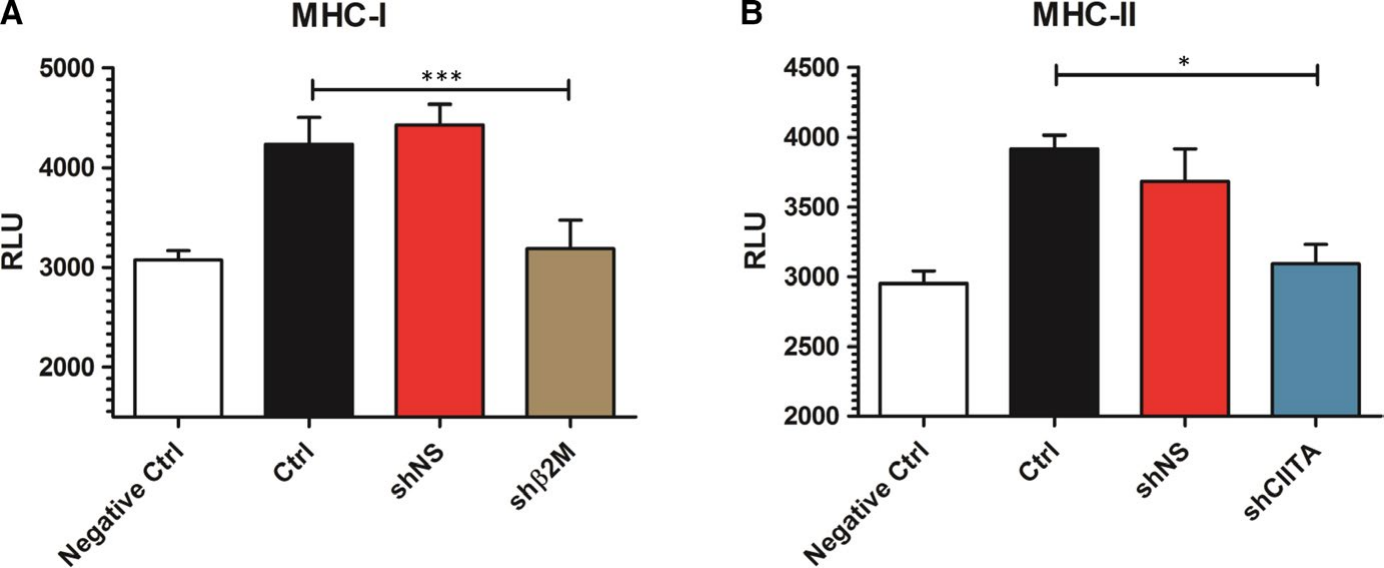
SLA silencing prevents antibody‐mediated cellular‐dependent cytotoxic responses. The strength of humoural immune response was assessed by performing antibody‐mediated cell‐dependent cytotoxicity (ADCC) assays. The activation of Jurkat effector cells was measured by detecting bioluminescence activity after 6 h incubation with ICCs‐derived cells with and without anti‐SLA‐specific antibodies. Graph shows relative luminescence units (RLU) detected in ADCC assays after effector cell exposure to ICCs‐derived cells in the presence or absence of A, anti‐SLA class I or B, anti‐SLA class II‐specific antibodies. Graphs show means and standard deviations of four independent ADCC assays. Statistical significance was calculated using one‐way ANOVA followed by Tukey's post hoc test. *P < .05; ***P < .001

Pre‐formed or de novo produced xenoantibodies specific for SLA class I or SLA class II molecules represent a potential risk factor for the survival of the xenograft.^25^ In order to analyse the impact of SLA class I and SLA class II on xenogeneic humoural immune responses, antibody‐dependent cellular‐mediated cytotoxicity (ADCC) assays were performed (Figure 6). Co‐cultured SLA class I‐silenced ICC‐derived cells with anti‐SLA‐specific antibodies showed significantly (*P* < .001) decreased cytotoxicity levels (3188 ± 286 RLU) compared to the control NT cells (4236 ± 268 RLU; Figure 6A). Moreover, there was no significant difference in bioluminescence levels of effector cells towards shβ2M‐expressing targets compared to the negative control (3188 ± 286 to 3075 ± 90 RLU, respectively). Regarding SLA class II, co‐cultured shCIITA‐expressing cells with anti‐SLA class II‐specific antibodies showed a significant (*P* < .05) decrease in cytotoxicity levels compared to the SLA class II‐expressing ICC‐derived cells, 3098 ± 274 to 3915 ± 196 RLU, respectively (Figure 6B). In addition, cytotoxic levels obtained from shCIITA cells (3098 ± 274) did not show any significant difference to negative control (2951 ± 153). Cytotoxicity levels detected in cultures using shNS‐expressing or control cells were similar, in the presence of either anti‐SLA class I or anti‐SLA class II antibodies. These results show that silencing SLA class I or SLA class II reduces xenogeneic ADCC immune responses of the recipient towards the graft.

### 3.7 Reassembling of genetically engineered SLA‐silenced Islets

**Figure 7:**
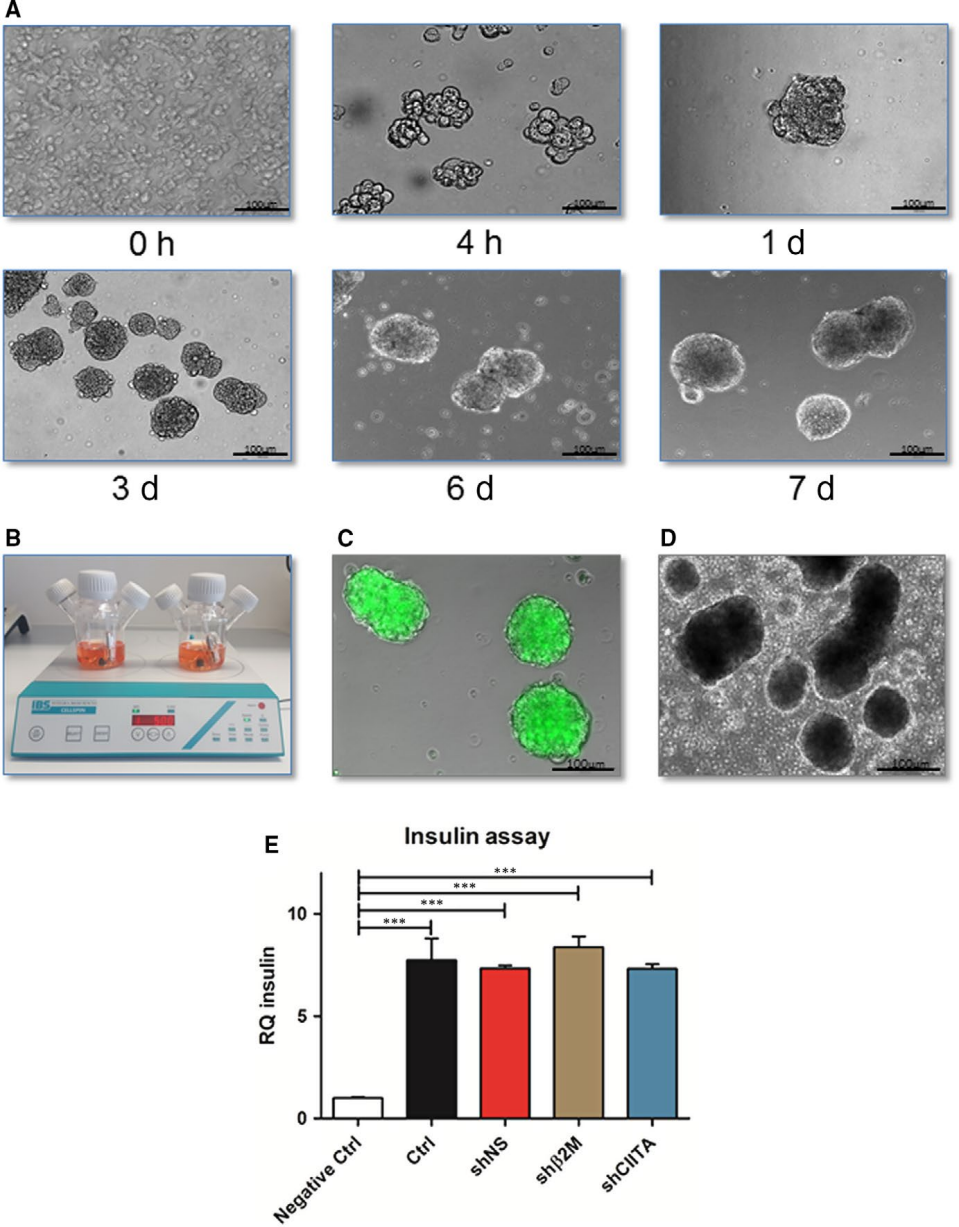
Reassembling of functional low immunogenic ICCs after single‐cell engineering. Genetically engineered cells were cultured for 7 d in a stirred bioreactor flask in the presence of collagen type VI to reassemble the ICCs. A, Representative microscopy images at different time points of the reassembling of the genetically engineered islets. B, Stirred bioreactor flasks containing genetically engineered ICCs‐derived cells during the reassembly of the ICCs. C, Representative microscopy image of the genetically engineered ICCs expressing GFP at the final stage of the islet reassembling. D, Illustrative microscopy image of native non‐engineered islets. Scale bar 100 µm. E, Reassembled ICCs were handpicked and incubated without (Negative Ctrl) or with 20 mmol/L glucose for 1 h at 37°C. Non‐engineered reassembled ICCs (Ctrl) or shRNA‐expressing reassembled ICCs were collected after glucose treatment, and total RNA was isolated for insulin transcript levels analysis. Graph shows insulin transcript levels of reassembled ICCs before and after stimulation with glucose. Insulin transcript levels were normalized to the endogenous control gene GAPDH. Graph shows means and standard deviations of three independent assays from all different ICCs. Statistical significances were calculated using one‐way ANOVA followed by Tukey's post hoc test. ***P < .001

We designed a multistep approach to reduce the immunogenicity of pig pancreatic islets without compromising their functionality. To achieve complete genetic engineering of the islets, they were enzymatically dissociated into single cells. Using stirred bioreactor flasks, SLA‐silenced islet‐like clusters were reassembled within 7 days of culture with the support of collagen VI (Figure 7A,B). Reassembled islets constituted by GFP‐expressing and SLA‐silenced genetically engineered ICCs‐derived cells showed the typical pancreatic islet morphology as observed in native non‐engineered islets (Figure 7C,D). These results show the feasibility to reassemble ICCs‐derived cells to their original 3D configuration after single‐cell genetic engineering.

### 3.8 Genetically engineered islets remain functional after reassembling

We investigated the effects of lentiviral transduction and silencing SLA expression on cell functionality after islet reassembling. After genetically engineered cells had been reassembled into their original 3D islet configuration, a glucose‐stimulated insulin secretion (GSIS) assay was performed with and without stimulation with glucose (Figure 7E). Addition of glucose significantly (*P* < .001) enhanced insulin transcript levels to 7.7 ± 1.0‐fold in control cells, 7.3 ± 0.2‐fold in shNS, 8.4 ± 0.5‐fold in shβ2M and 7.3 ± 0.2‐fold in shCIITA compared to 1 ± 0.1‐fold in islet cells cultures where no glucose was used. Remarkably, genetically engineered islets demonstrated similar responsiveness to glucose stimulation when compared to control islets.

## 4 DISCUSSION

Xenotransplantation has the potential to overcome the severe shortage of human organ donors. Recent studies suggest that pancreatic islet xenotransplantation has an impressive potential to become the first standardized clinical application of xenotransplantation.^26^, ^27^

However, the high immunogenicity of the grafts poses a major obstacle for the success and widespread of xenotransplantation as a standard therapy. Porcine xenografts are targets for pre‐formed anti‐pig antibodies contributing to complement cascade activation and finally to hyperacute rejection.^28^ Global research efforts have generated significant advances in the development of genetically engineered pigs to prevent hyperacute and acute rejection. A combination of alpha‐GAL gene knockout with expression of human complement‐regulatory genes such as CD46 (membrane cofactor protein), CD55 (decay‐accelerating factor) and CD59 (protein or membrane inhibitor of reactive lysis) and thrombomodulin has shown promising results in the reduction of the coagulation and inflammation in the xenograft.^29^, ^30^, ^31^ Furthermore, acute rejection mediated by the concerted action of T cells, B cells and antibodies may lead to xenograft dysfunction and rejection. After xenotransplantation, MHC mismatch between donor and recipient leads to humoural and cellular immune responses, ultimately leading to the development of acute and chronic rejection of the graft.^32^, ^33^ Although strong immunosuppressive treatments may prevent or delay acute rejection, the development of chronic vasculopathies is a risk for the survival of the xenograft.^28^

Previous studies from our laboratory demonstrated that silencing MHC expression on cells and tissues contributes to preventing cellular and humoural immune responses and supporting graft survival in an allogeneic setting.^16^, ^34^, ^35^, ^36^ Hence, here we evaluated the impact of silencing SLA expression in the strength of human humoural and cellular immune responses. As the compact structure of the islets represents a major hurdle to vector transduction of the inner cell mass of the islets, we have dissociated the islets to achieve higher transduction efficiencies and allowing the generation of low immunogenic islets. According to the limited data available in the literature, islet isolation from neonatal pigs may have advantages over adult pigs. Some reports suggest that islets’ isolation during the first week of life may have minor advantages over later weeks. Among the advantages listed, the authors highlight the surprisingly better islet yield per gram of pancreas even when compared to adult pigs, together with an easier islet isolation and higher resistance to hypoxia, leading to increased islet quality after isolation.^37^, ^38^, ^39^ Furthermore, in the present study, a third‐generation lentiviral vector was used to deliver shRNA targeting β2‐microglobulin and CIITA on neonatal ICCs‐derived cells to persistently reduce SLA class I and class II expression, respectively. Expression of the reporter gene GFP demonstrated high transduction efficiencies, showing a significant improvement compared to the direct transduction of the intact pancreatic islets as previously described.^22^ In addition to the reporter gene GFP, the significant downregulation of SLA class I and SLA class II at mRNA and at protein levels confirmed the efficient transduction of ICCs‐derived cells. During inflammatory responses, IFN‐γ and TNF‐α are frequently up‐regulated and have the capability to regulate the expression of several genes such as MHC.^40^, ^41^ Increased expression of MHC class I and class II enhanced immunogenicity of the graft after transplantation.^42^ In this study, we used IFN‐γ and TNF‐α to mimic an inflammatory environment, but without inducing cell necrosis. Under IFN‐γ and TNF‐α stimulation, ICCs‐derived cells showed a significantly lower increase in SLA‐I and SLA‐II expression levels in comparison with non‐engineered or shNS‐engineered ICCs‐derived cells. This demonstrates an efficient shRNA‐mediated regulation of gene expression even under a strong inflammatory environment.

Previous studies had demonstrated induction of human T cell activation by the interaction with SLA class I and class II.^32^, ^43^ T cell activation can be induced by indirect binding of T cell receptors to antigen‐presenting cells of the host expressing MHCs or directly binding T cell receptors to SLA class I and class II of porcine cells. Even though we were not able to demonstrate a significant difference in T cell degranulation towards SLA class I‐silenced cells, the co‐culture of SLA class II‐silenced ICCs‐derived cells with T cells revealed significant reduced cytotoxicity when compared to the NT cells suggesting that downregulation of SLA class II may provide protective effect against T cell xenogeneic immune responses.

Insufficient inhibitory interactions to balance interactions with activating ligands can lead to destruction of the xenogeneic graft.^44^ Here, no increase in NK cell degranulation towards SLA‐I‐silenced cells was observed. Surprisingly, we found a significantly reduced NK cell cytotoxicity, suggesting that silencing SLA class I molecules may lead to protection of the graft. However, further studies on the mechanism of NK cell regulation in a xenogeneic environment are required to fully understand this effect.

In a xenotransplantation setting, a delayed antibody‐mediated response is associated with the production of anti‐pig antibodies, which may lead to injury caused by complement molecules or immune cells, such as T cells, NK cells or macrophages.^45^ Remarkably, a significantly decreased cytotoxicity was observed when SLA class I‐silenced cells were co‐cultured with anti‐SLA‐specific antibodies. This suggests that SLA class I‐silenced ICCs‐derived cells induce a weaker xenogeneic humoural immune response in comparison with fully SLA‐I‐expressing cells. In addition, SLA class II‐silenced cells showed reduced cytotoxicity to similar levels as the negative controls, where no antibody was used, suggesting that SLA class II‐silenced cells are protected against xenogeneic humoural immune responses.

Pancreatic islet transplantation is a promising therapy to cure patients with type I diabetes. The reassembly of porcine genetically engineered islets might provide an important source to overcome critical immunological barriers. Llacua et al revealed a positive influence of collagen type VI on human islet survival in immune‐isolating microcapsules.^46^ In this study, we demonstrated the feasibility to reassemble genetically engineered islets in a 7‐day protocol using collagen type VI to improve islet reassembly. Collagen is a versatile material that has been broadly applied in regenerative medicine and as a drug vehicle.^47^ Collagen VI improves survival and functionality of reassembled pig islets by providing the required extracellular matrix and supporting the stability of the islet 3D structure. Since dissociated single β‐cells are not capable of secreting insulin in vitro,^48^, ^49^ we assessed the insulin transcript levels of the reassembled islets upon glucose stimuli. We found that insulin expression was seven‐fold to eight‐fold higher under glucose stimuli compared to medium without glucose, suggesting that reassembled genetically engineered islets maintained their ability to “sense” glucose and as a consequence up‐regulate insulin expression. The reassembly of the islets using stirred bioreactor flasks and the observed glucose‐dependent insulin secretion of these “neo‐islets” offer the possibility to perform a large‐scale reassembling and may become an additional tool to fulfil the need of high amounts of islets to maintain normoglycemia in an adult human.

Despite these encouraging results, our study possesses some limitations. One important limitation is that we are still in an early in vitro phase, and the function and capability to escape the immune response of the reassembled islets need to be tested in a transplantation model. Another possible limitation may be associated with the amount of β‐cells that can be isolated from neonatal pigs. Despite neonatal pigs being the best option available, the number of cells necessary to fulfil the needs of an adult human being might be challenging to accomplish and further technological development regarding cell expansion may be necessary.

Here, we have demonstrated the feasibility to efficiently generate “immunologically invisible” ICCs by silencing SLA expression after single‐cell genetic engineering. The use of lentiviral vectors enables permanent or conditional gene expression regulation of key genes, in particular MHC class I and class II. Silencing of MHC class I and class II molecules on ICCs‐derived cells from pigs, with further reassembly into islet‐like 3D configuration, might represent a promising approach to improve graft survival in a xenotransplantation setting, reducing the needs of strong immunosuppressive therapies and without compromising β‐cell function.

## CONFLICT OF INTEREST

The authors confirm that there are no conflicts of interest.

## AUTHOR CONTRIBUTION

MCO designed, performed experiments, analysed the data and contributed to write the manuscript. EV, MV, YY, HJS and OP performed experiments and analysed the data. HN, RS, BP and JS contributed with crucial material and advice. RB analysed the data and contributed with advice. CF designed and supervised the study, analysed the data and contributed to write the manuscript.

## Data Availability

The data that support the findings described in this study are available in the article.
